# Chronic Suppurative Otitis Media: A Comprehensive Review of Epidemiology, Pathogenesis, Microbiology, and Complications

**DOI:** 10.7759/cureus.43729

**Published:** 2023-08-18

**Authors:** Mihika Khairkar, Prasad Deshmukh, Hindol Maity, Vijayshri Deotale

**Affiliations:** 1 Department of Otolaryngology, Head and Neck Surgery, Jawaharlal Nehru Medical College, Datta Meghe Institute of Higher Education and Research, Wardha, IND; 2 Department of Microbiology, Mahatma Gandhi Institute of Medical Sciences, Sevagram, Wardha, IND; 3 Department of Pathology, Jawaharlal Nehru Medical College, Datta Meghe Institute of Higher Education and Research, Wardha, IND

**Keywords:** tympanoplasty, cholesteatoma, biofilm infections, aom, csom

## Abstract

Otitis media is a significant contributor to healthcare visits and the prescription of drugs. Its associated complications and consequences pose the primary factors for preventable hearing impairment, especially in developing nations. Chronic suppurative otitis media (CSOM) is prevalent among children globally as one of the commonest chronic infectious diseases during childhood. The subsequent complications and sequelae play a central role in causing avoidable hearing loss, particularly within developing countries. In addition to impaired hearing, this condition can lead to severe health complications, such as issues involving the intracranial region. Despite the involvement of microbial, immunological, and genetic factors as well as Eustachian tube characteristics, in the development of CSOM, there remains a need for further elucidation regarding its pathogenesis. Based on its microorganisms, the treatment of choice will be affected to prevent further complications in the child. The primary approach to treating acute otitis media (AOM) involves effectively addressing ear pain and fever symptoms, while antibiotics are only administered in cases where children experience severe, long-lasting, or frequent infections.

Despite the extensive investigation on AOM pathogenesis, research is scarce regarding CSOM. Given that antibiotic resistance and drug-induced ear damage are growing problems and surgery-related complications, it is imperative to devise effective therapeutic interventions against CSOM arises. Therefore, comprehending the host's immune function concerning CSOM and identifying how bacteria sidestep these potent responses becomes crucial. Acquiring insight into molecular mechanisms associated with CSOM will enable scientists to formulate innovative treatment approaches to combat this disease, thereby averting hearing loss consequences. The management consists of watchful waiting, primarily for children with chronic effusions and hearing loss.

## Introduction and background

Chronic suppurative otitis media (CSOM) is one of the most common infectious diseases encountered in the practice of otolaryngology, affecting children globally, regardless of geographical or socioeconomic status. The incidence of CSOM is estimated at more than 20 million people worldwide. Research conducted in industrialized nations reveals that roughly 80% of preschoolers might have gone through at least a single episode of acute otitis media (AOM) before they turn three, and nearly 40% will experience six or more recurrences by the time they reach the age of seven. Comparable to other communicable ailments, AOM's impact varies significantly among different countries based on factors such as suppurative complications prevalence like mastoiditis, meningitis incidence rate, and sequelae development likelihood associated with CSOM resulting in hearing loss [1-3]. CSOM is a condition where there is a prolonged inflammation of the mucosa in the middle ear and mastoid space. It persists for more than two months, which causes a hole to form in the eardrum and results in ongoing discharge from the ear canal. This persistent ailment can cause profound health implications, such as complications within the intracranial area and significant morbidity among those affected. These adverse outcomes make CSOM a major public health issue that requires immediate attention to curb its prevalence worldwide [4-7]. The occurrence of this condition exhibits considerable variation across different nations; however, it is particularly prevalent in countries with low- and middle-income levels. By definition, chronic suppurative otitis media is a chronic infectious disease associated with inflammation of the middle ear and mastoid mucosa, which can lead to perforation or formation of a tympanostomy tube and discharge (otorrhea) [8,9]. There is no common consensus regarding symptom duration, although some classifications define it as "otorrhea through a perforated tympanic membrane lasting for at least two weeks," whereas others describe 'chronic' symptoms persisting beyond six weeks [10-13].

The definition of CSOM varies depending on the duration and severity of symptoms, but it is generally acknowledged that CSOM follows unsuccessfully or partially treated acute otitis media [14,15]. However, there is no clear distinction between otorrhea as a sign of AOM and CSOM. It should be noted that tympanostomy tube otorrhea, which results from complications with placing ear tubes, should not be confused with CSOM. Chronic otitis media with effusion (OME) without active infection or perforation in the eardrum must also be differentiated from CSOM and chronic perforations without middle-ear infections. When dealing with cases involving cholesteatoma deposition around the inflamed tissues inside the ears, it is referred to as CSOM with cholesteatoma. However, CSOM primarily affects children in the first five years of life, particularly in developing countries and among populations with craniofacial anomalies [16].

CSOM has two distinct types: the first is the benign or tubotympanic type, which primarily affects the inferior anterior region of the middle ear cleft and results in permanent central perforation. Despite this symptom, individuals with this type of CSOM are not prone to severe complications. On the other hand, the second classification is known as the malignant or atticoantral type-also called the "danger" type since it involves both the attic and posterosuperior regions of the middle ear-posing severe health risks for those affected by it [17]. The misuse and excessive use of antibiotics in recent times have led to changes in important bacterial strains as well as their responses to antibiotics. As a result, addressing this situation has become more complex. There have been reports on the changing prevalence and antibiogram (antibiotic sensitivity patterns) of micro-organisms causing chronic otitis media over time and across different regions. This is likely due to the indiscriminate usage of antibiotics. Therefore, regularly updating information on the prevalence and antibiogram of these microorganisms for chronic otitis media would aid in patient treatment. Currently, early identification through microbiological diagnosis ensures timely and effective treatment while also helping prevent complications.

## Review

Risk factors

Several risk factors can contribute to the development of CSOM. It is more commonly observed in children, especially those in lower socioeconomic groups. The immature immune system and higher susceptibility to infections in children increase their risk. Other risk factors include frequent or untreated acute otitis media, inappropriate treatment for ear infections, poor hygiene, and environmental factors such as living in an unclean or overcrowded environment, low socioeconomic status, limited access to healthcare facilities, inadequate healthcare resources, genetic predisposition, malnutrition, and structural abnormalities like cleft palate [18]. Other unique factors that increase the likelihood of CSOM include frequent AOM and parents who have had CSOM in the past [3]. Further, allergies can be considered another risk factor, given that certain research studies have indicated the existence of allergens causing hindrance to both the Eustachian tube and nasal passages [19].

Methodology

Literature Search

To gather extensive information on the topic, we performed a thorough literature search with 47 PDF-accessible articles after removing the duplicates published after 2015 (Appendix, Table 1). Additional articles were referred to, which were cited by these articles. We utilized various databases such as PubMed, Cochrane Library, and Google Scholar. Our search included specific keywords relevant to our study, including "chronic suppurative otitis media", "CSOM", "microbiology", "treatment", "prevalence", and "burden of disease". We also manually searched the reference lists of relevant articles to identify additional studies.

Inclusion and Exclusion Criteria

Incorporated in our research were studies that explored the various aspects of CSOM, such as microbiology, epidemiology, diagnosis, treatment, and prevention. However, we opted to exclude non-English studies and those that did not undergo the peer-review process.

Data Extraction and Synthesis

In order to conduct our analysis, we obtained information from each study that was included in the research. To evaluate this collected information effectively, we employed a narrative approach, which allowed us to provide an overview while highlighting important and current details.

Data Analysis

In our analysis, we employed a qualitative method to examine the data. We focused on identifying recurring themes and patterns that emerged from the studies included in our research. Furthermore, we utilized descriptive statistics as a means of succinctly summarizing the findings obtained.

Epidemiology

CSOM is frequent, especially prevalent in underprivileged communities and developing nations. Its predominance varies between 1 and 46% according to the demographic region and population [20]. Although CSOM affects adults too, it primarily strikes children, mainly from two- to five-year-olds. It has also been documented among elders more frequently [21]. A shared characteristic of these congenital abnormalities pertains to insufficient Eustachian tube functionality, a condition that elevates the vulnerability of affected children towards middle-ear ailments [22]. Risk determinants primarily related to CSOM consist of (A) frequent occurrences of acute otitis media, (B) infections within the upper respiratory tracts, (C) any injury affecting the tympanic membrane, and (D) inadequate nutrition and poor living arrangements.

Globally, the illness impacts many individuals, ranging from 65 to 330 million. The majority who are affected reside in less developed nations. It has been approximated that there is an annual incidence of 31 million new cases of CSOM, out of which approximately one-fifth account for children below five years old [23].

Mechanisms/Pathophysiology

**Figure 1:**
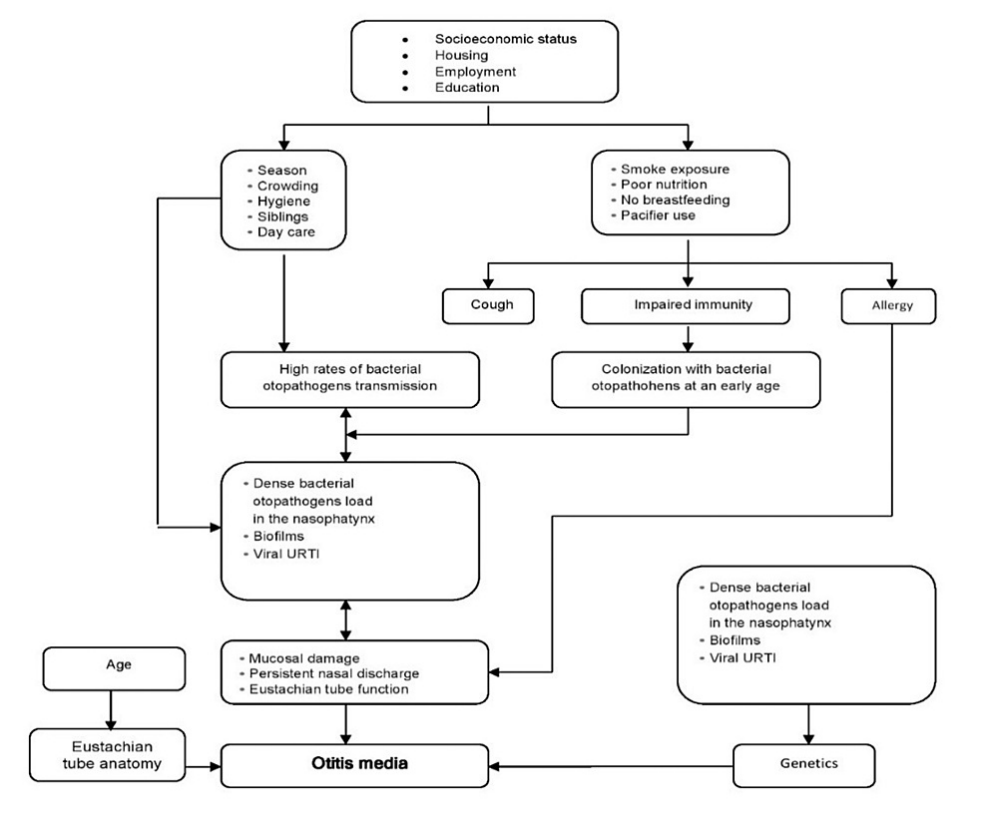
Pathways and factors affecting otitis media. Image adapted with permission from Reference [25].

Despite the substantial disease burden, OM cases in developed countries are typically uncomplicated and self-limiting, with little incidence of persistent hearing impairment or developmental delay [24]. However, high-risk populations residing in developing and well-developed regions often experience significant lifelong hearing loss due to complex exposure to multiple genetic, environmental, and social risk factors. The progression of OM pathology is an intricate amalgamation that begins with early colonization by bacteria within the nasopharynx, followed by premature onset acute otitis media (AOM). Over time, this leads to acute inflammation cycles within the middle ear due to ongoing exposure to infective agents such as bacterial persistence through biofilm formation; viral infection ultimately results in severe chronic conditions involving the ears (Figure 1) [25].

Eustachian Tube Anatomy

A functional Eustachian tube plays a vital role in safeguarding the middle ear by preventing the invasion of bacterial otopathogens and respiratory viruses, facilitating the drainage of secretions from this area, and ensuring pressure equalization. In infants, immature Eustachian tube anatomy significantly contributes to susceptibility to infections in the middle ear. The main line of defense against entry and colonization by otopathogens is located within the epithelium lining this structure, consisting primarily of ciliated respiratory epithelial cells that synthesize antimicrobial proteins (e.g., lysozyme). Additionally, goblet cells interspersed among these produce both serous and mucoid mucus. The action controls the proper direction through which mucociliary flow occurs, moving from the nasopharynx toward the middle ear, while antimicrobial protein secretion protects against inflammatory reactions caused by bacteria residing there [26].

In neonates and young children less than one year of age, the Eustachian tube is characterized by a shorter length, a wider diameter, and a greater horizontal inclination. This structure permits easier transmission of pathogens to the middle ear, leading to a higher susceptibility to otitis media. Placing infants in a supine position also aggravates their vulnerability to infections. As kids grow older, a shift downward in skull base elevation results in a gradual increase at an angle from 10° (at birth) up to about 45° (in adults), while concomitantly increasing in length from approximately 13 mm at birth until it reaches ∼35 mm when fully grown-up. Adjustments occurring due to these alterations, along with maturation in immune function, can contribute towards a decreased risk profile about OM even among those classified as high-risk candidates for otitis media infection [27].

Bacterial Infections and Biofilms

The pathogenesis of CSOM involves a fusion of various factors that contribute to the continued existence of middle ear infections and inflammation. The onset of this condition generally starts with acute otitis media, characterized by an infection in the intermediate ear. Inadequate treatment or inherent risk elements may result in the development of CSOM, an advanced stage of AOM. Impaired Eustachian tube function plays a significant role in leading to CSOM. When a rupture in the tympanic membrane occurs spontaneously or due to the insertion of tubes into the eardrum, it leads to the loss of the middle ear's "gas cushion." This results in backflow or reflux of secretions from the nasopharynx through the Eustachian tube [28].

As a consequence, contamination with respiratory pathogens takes place. Pressure equilibrium between the middle ears and the outer atmosphere can lead to insufficient drainage and ventilation, causing bacteria or fungus overgrowth. Reduced ciliary functions of the middle ear and Eustachian tubes; persistence or frequent recurrence is another significant factor leading to chronic irritation among individuals with CSOM; it could be due to incomplete eradication methods against bacterial pathogens or restrained immune response mechanisms inadequately combating infections. Biofilms in CSOM, comprising structured bacterial communities enclosed within a protective matrix, can be attributed to the persistence of the infection [29,30]. The presence of biofilms enhances antibiotic resistance and the immune evasion ability of bacteria, thereby hindering effective treatment measures [31].

Microbiology

The microbiology of CSOM entails the existence of diverse microorganisms, occasionally including fungi, within the middle ear. Among these bacteria, *Pseudomonas aeruginosa* prevails and is deemed clinically consequential due to its adeptness in developing biofilms and capacity for antibiotic resistance. Alongside it, *Staphylococcus aureus* usually cohabits with a similar inflammatory impact, as this bacterium can generate harmful toxins that trigger tissue damage. Moreover, *Streptococcus pneumoniae* is another common cause of acute otitis media that may also lead to CSOM cases.

Other bacteria include *Haemophilus influenzae, Moraxella catarrhalis, Proteus *spp., and anaerobic microorganisms such as *Peptostreptococcus *spp. and *Prevotella *spp. Furthermore, in areas with specific geographic conditions or among individuals who have a weakened immune system, fungal infections like *Aspergillus *spp. and *Candida *spp. can also contribute to the condition's progression. To find out the anaerobic etiological agent, the otolaryngologist needs to remember the probability that anaerobic organisms are also causative agents. Based on the gram staining picture, the microbiologist can report the probable causative agent. Depending on factors like geographical location, personal immunity status, history of antibiotic use, and other host elements involved, CSOM microbiology varies greatly. Henceforth, appropriately identifying pathogens through microbiological testing is crucial for formulating effective treatment strategies to manage them effectively.

Immunology and Genetics

The body's immune system and genetic makeup are critical in managing CSOM. Immunoglobulins IgG, IgA, and secretory IgA are most effective in guarding against mucosal infections like CSOM. Locally synthesized by plasma cells within the middle-ear cavity mucosa, SIgA helps prevent bacterial attachment to and colonization of this area. However, children with CSOM may have deficient levels of SIgA [32]. The IgG-class immunoglobulin concentration primarily acts to facilitate phagocytosis either directly or indirectly via complement activation, which depends on the age factor. Children experiencing recurring upper respiratory tract illnesses portray reduced levels of specific subclasses, such as mostly low-targeting antibody activity from subclass-IgG2 in 10-20% of cases [33,34]. For antibodies to be action-ready for infection prevention, coating on bacteria walls stands prerequisite. While intense SIgA and IgG coatings prevail commonly during a case involving bacterial causation other than *Pseudomonas aeruginosa* responsible, there has been no sighted coverage when that particular pathogen causes an infection, indicating its resistance towards these two responses.

Despite conflicting data regarding inadequate antibody responses to otopathogens in children at risk of developing OM, there is increasing clarity on the possible involvement of cell-mediated impairment. The impact of genetics on these findings remains uncertain, and it is plausible that interactions between pathogens, hosts, and surroundings could play a part. Additional investigation is required for a comprehensive comprehension of how genetic factors contribute to the development of OM.

Complications and sequelae

**Figure 2:**
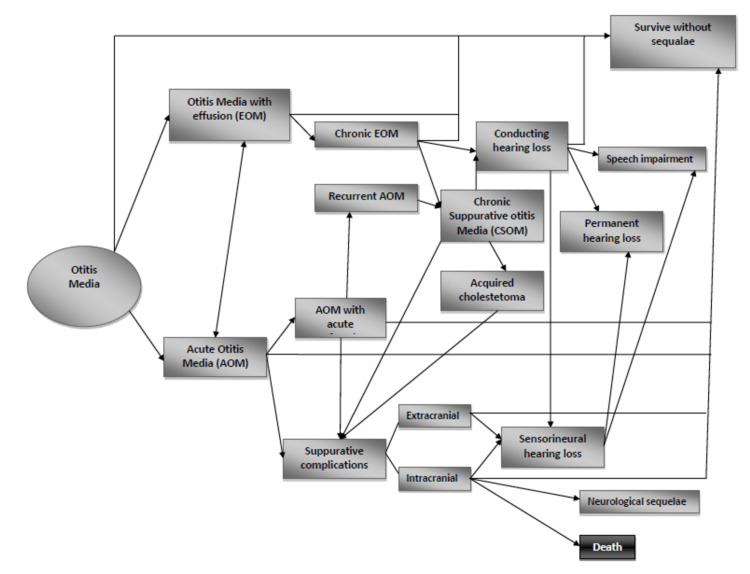
Sequalae of otitis media. Image adapted from Reference [10], which was originally published and distributed under the terms of the Creative Commons Attribution License, which permits unrestricted use, distribution, and reproduction in any medium, provided the original author and source are credited.

CSOM can result in various complications, including conductive or sensorineural hearing loss, extracranial complications (e.g., facial paralysis, subperiosteal abscess, mastoiditis), and intracranial complications (e.g., meningitis, cerebral abscess). Prompt diagnosis and management are crucial to prevent long-term consequences, particularly in pediatric patients. Meningitis and brain abscesses are the primary causes of death resulting from OM and CSOM. WHO estimates show that a significant number, ranging from 65 million to 330 million individuals, display symptoms associated with CSOM [23], out of which half suffer from hearing impairment. In developed nations, after hypertension and arthropathy, hearing loss (including conductive and sensorineural) is considered the third chronic ailment prevailing among older adults affecting their physical and mental well-being in considerable ways. Conversely, data on less industrialized countries' adult populations remains sparse regarding such conditions. Therefore, a thorough understanding of AOM's incidence rate across demographics must be established to reduce health issues alongside social-economic adversity by intervening at the right time (Figure 2). 

Prognosis

In general, CSOM’s outlook is favorable, provided it receives proper treatment and no complications are involved. A few cases may be refractory, which entails a more comprehensive assessment and management. It is vital to identify and treat the bacterial origin of acute otitis media, as this can lead to CSOM in most instances. The use of the Pneumococcus vaccine has resulted in a positive reduction in the incidence of acute otitis media, thereby decreasing the occurrence rate of patients presenting with CSOM.

Prevention

Due to the complex nature of OM, there are several preventive measures that can be employed. These measures primarily center around minimizing modifiable factors that contribute to risk, such as infectious agents and environmental hazards.

Vaccines directed against bacterial otopathogens: The vaccines aim to reduce or eliminate nasopharyngeal colonization by *S. pneumoniae* and *H. influenzae*. The vaccine has been added to the primary series of universal vaccination schedules with a 20% reduction in the CSOM by using ventilation tubes.

Deterrence and patient education

Parents need to be informed and guided about the significance of consistent visits for their child's overall health check-ups, as well as being encouraged to promptly seek medical attention when their children report ear pain or discomfort. Additionally, it is crucial to take into account any concerns raised by teachers regarding potential hearing loss in a child. Addressing and managing CSOM in a timely manner is critical in reducing the risk of long-term complications that may impact the child's well-being.

Enhancing Healthcare Team Outcomes

CSOM is a medical condition characterized by a persistent infection in the middle ear that damages the eardrum. This disease usually manifests during early childhood, typically around two years old, and may follow an episode of acute otitis media. It is vital to isolate the causative agent upon suspicion of this ailment promptly. If left unchecked, CSOM can result in severe complications such as polyps, sclerosis, or hardening of tissues within the ear cavity walls; tympanosclerosis or thickening and scarring on hearing-related components within our ears causing conductive hearing loss; labyrinthitis, which triggers inflammation affecting areas close to inner-ear nerves leading to balance issues with dizziness spinning sensations and nausea over time these symptoms lead up cognitive impedances like disorientation seizures, etc.; epidural abscesses culminating in excruciating headaches swelling nervousness convulsion depression (psychiatric disorders), subdural abscesses making it difficult for one's coordination, tongue movement awareness, memory processing, communication problem solving skills, mental flexibility, adaptation capability, along with development requiring surgery intervention if necessary, alongside brain abscesses compromising one's ability, even more debilitating auditory effects are so familiar that children are unable to attend school regularly because of decreased academic performance impacting scholastic ability.

Limitations

There are a number of limitations in our review that need to be acknowledged. Firstly, it is important to note that non-English studies were excluded from the analysis, which may lead to some bias and restrict the generalizability of our findings. Additionally, we should consider potential publication bias as another limitation since there is always a possibility that studies with significant results are more likely to be published compared to those with null or non-significant findings. Lastly, it's worth mentioning that the quality of the included studies varied across different sources, which could have an impact on the overall validity and reliability of our conclusions.

## Conclusions

CSOM remains a significant childhood chronic infectious disease with global implications. A child with persistent upper respiratory tract infection with pain in the ear has the highest chance of developing otitis media. If failed to treat timely can lead to severe complications. Understanding its epidemiology, pathogenesis, microbiology, and associated complications is essential for effectively managing and preventing long-term sequelae. The complications of CSOM do affect the cognitive and educational development of the child as well as long-term effects on the child's communication. Further research is needed to enhance our knowledge of CSOM and develop targeted interventions.

## Appendices

**Table 1:** Literature reviewed.

Paper title	Abstract summary	Authors	DOI	Year
Complications of chronic suppurative otitis media	Two hundred fifty patients of all age having complications due to chronic suppurative otitis media enrolled from admitted patients in ENT department Khyber Teaching Hospital Peshawar were included in the study.	Hasan SZ, Gul I, Ahmad A	10.37762/jgmds.3-01.46	2016
The comparative analysis of the clinical and morphological picture of the various forms of chronic suppurative otitis media	The surgical intervention on the dry ear of the patients with the tubotympanic form of chronic suppurative otitis media was 91.4%.	Baike EV	10.17116/otorino201681230-33	2016
Management of acute otitis media in children six months of age and older	A bulging tympanic membrane is a major diagnostic criterion for acute otitis media.	Le Saux N, Robinson JL	10.1093/PCH/21.1.39	2016
Otitis media with effusion: comparative effectiveness of treatments	Adjuvant adenoidectomy at the time of initial grommet insertion decreased the risks of reinsertion and re-admission for conditions related chronic otitis media with effusion.	Karuna N, Sreekanth G	10.9790/0853-150722934	2016
The intraoperative pathological findings in cases of chronic suppurative otitis media with central perforation of tympanic membrane at a tertiary care center in Eastern Nepal	Cholesteatoma can be found in tubotympanic type of chronic suppurative otitis media.	Thakur S, Ghimire N, Acharya R, Singh S, Afaque A	10.3126/AJMS.V8I1.15944	2017
Mycological profile of chronic suppurative otitis media in a tertiary care hospital in South India	The irrational use of broad spectrum antibiotics, use of steroids and immunodeficiency disorders favors the secondary infection by fungi.	Haneefa S, Bhama S, Rajahamsan J	10.20546/IJCMAS.2017.602.018	2017
Bacteriological profile of chronic suppurative otitis media in a tertiary care hospital	Treating active discharge of chronic suppurative otitis media reduces the bacterial load in the middle ear.	Sowmya TR, Dudda R, Prasad M, Balaji NK, Sumangala B, Gudikote M	10.18203/ISSN.2454-5929.IJOHNS20173031	2017
Aetiological factor for chronic suppurative otitis media: a retrospective study	Chronic suppurative otitis media is an important cause of morbidity in very large group of Indian population.	Hardik D, Sinha M	10.18203/ISSN.2454-5929.IJOHNS20170931	2017
Noninvasive in vivo optical coherence tomography tracking of chronic otitis media in pediatric subjects after surgical intervention	Surgical intervention consisting of myringotomy and tympanostomy tube placement provides a means to clear the middle ear of infection-related components.	Guillermo LM, Pande P, Nolan RM, Shelton RL, Porter RG, Novak M, Spillman D, Chaney E, McCormick D, Boppart S	10.1117/1.JBO.22.12.121614	2017
Panel 7: Otitis media: treatment and complications	Watchful waiting is optional in mild to moderate acute otitis media.	Schilder AG, Marom T, Bhutta MF, Casselbrant ML, Coates H, Gisselsson-Solén M, Hall AJ, Marchisio P, Ruohola A, Venekamp RP, Mandel EM	10.1177/0194599816633697	2017
Acute otitis media-an update	Acute otitis media can be defined as a rapid onset of fever and otalgia.	Shawabka MA, Haidar H, Larem A, Aboul-Mahmood Z, Alsaadi A	10.15406/JOENTR.2017.08.00252	2017
Spectrum and antibiogram of bacteria in chronic suppurative otitis media and biofilm formation	Samples of pus were collected from the deeper aspect of external auditory meatus.	Asima B, Samhitha V, Karthik S	10.15406/JSRT.2017.02.00069	2017
Microbiological profile of chronic suppurative otitis media	Chronic suppurative otitis media is a major problem in developing countries like India.	Ghogare H, Vitore V, Hatkar S, Bhalchandra MH, Wyawhare AS, Bansal VP	10.20546/ijcmas.2018.710.128	2018
Comment to empirical therapy for chronic suppurative otitis media	Culture and sensitivity is essential in eradicating the causative organism.	Bareeqa SB, Ahmed SI	10.1177/1179550618810226	2018
The medical assistance seeking behaviour of the patients presenting with suppurative chronic otitis media and their treatment in Moscow	The most frequently practiced form of the surgical treatment employed for the management of suppurative chronic otitis media is tympanoplasty.	Garov EV, Khamzalieva RB, Zelenkova VN, Garova EE, Meparishvili AS, Lapenko EG	10.17116/otorino20188305126	2018
Antimicrobial susceptibility pattern of bacterial isolates in patients of chronic suppurative otitis media in a tertiary care hospital in India	The most common organism isolated in patients suffering from chronic suppurative otitis media was *Pseudomonas* spp.	Mahajan RK, Agarwal S, Jeram H, Vashishtha RC	10.18203/2320-6012.IJRMS20184434	2018
A study of aerobic bacteriological profile of chronic suppurative otitis media in a tertiary care hospital, South India	Staphylococcus isolates were highly sensitive to vancomycin and linezolid.	Smitha NR, Jnaneshwara KB, Patil AB, Harshika YK, Medegar S	10.18231/2394-5478.2018.0096	2018
Surgical treatment of chronic ear disease in remote or resource-constrained environments.	Tympanoplasty for subtotal wet perforations is frequently encountered in resource-poor conditions.	Smith MC, Huins C, Bhutta M	10.1017/S0022215118002165	2018
Role of computed tomography in diagnosis of complications in chronic suppurative otitis media	CT scans are used to diagnose complications in chronic suppurative otitis media among 40 patients between June 2018 and June 2019.	Abdulsattar OA, Alturaihy SH, Hussein AA	10.36295/asro.2019.22052	2019
A rare case of odontogenic chronic suppurative otitis media	A dental abscess was found on computed tomography to be the source of concurrent ipsilateral maxillary sinusitis and mastoiditis.	Schartz DA, Polacco MA, Holmgren EP, McCool RR	10.7759/cureus.4284	2019
Complications of chronic suppurative otitis media and their management: five year study at tertiary care centre	The complications of chronic suppurative otitis media are a great challenge in developing countries.	Mohite AA, Mane RS, Patil BC, Mohanty RM	10.18203/ISSN.2454-5929.IJOHNS20192593	2019
Aerobic bacteriological profile of chronic suppurative otitis media and special reference to methicillin resistant *Staphylococcus aureus*	The most acceptable definition of chronic suppurative otitis media is an infection of the middle ear that lasts more than three months and is accompanied by tympanic membrane perforation.	Hundekar JR, Surekha YA, Krishna S	10.20546/IJCMAS.2019.807.257	2019
Prevalence and etiological agents for chronic suppurative otitis media in a tertiary hospital in Tanzania	The most common isolates were facultative anaerobes.	Abraham ZS, Ntunaguzi D, Kahinga AA, Mapondella KB, Massawe ER, Nkuwi EJ, Nkya A	10.1186/s13104-019-4483-x	2019
Sinonasal and nasopharyngeal pathology in chronic otitis media patients: a prospective study	The concept of a relationship between sinonasal and nasopharyngeal pathologies and chronic otitis media has been supported by the present study.	Kayedjohar KR	10.17511/jooo.2019.i07.02	2019
Causality of chronic suppurative otitis media: an observational study	*Pseudomonas aeruginosa* was the most common causative agent of chronic suppurative otitis media at our institute.	Ayaz Z, Taj B, Yaseen MS, Ishaq U, Laique T, Malik J, Baig A, Sakhawat K	10.7759/cureus.9832	2020
Evaluation of single stage tympanomastoid surgery in unsafe chronic suppurative otitis media in Kashmiri population: a cross sectional study	The development and appropriate use of antibiotics have led to decrease in potentially devastating complications.	Mir P, Makhdoomi O, Syed WA	10.18203/issn.2454-5929.ijohns20201691	2020
Relation between the duration of disease and audiogram findings in tubotympanic type of chronic suppurative otitis media after myringoplasty	Chronic suppurative otitis media is defined as a chronic inflammation of the middle ear and mastoid cavity which presents with recurrent ear discharges through a tympanic perforation.	Rajamohan G, Saai RT	10.18203/issn.2454-5929.ijohns20205061	2020
A study on chronic suppurative otitis media in a Tribal Area Medical College	Chronic suppurative otitis media is a very common disease in all age group and both sexes.	Satyajit M	10.20546/IJCMAS.2020.912.220	2020
A study of complications of chronic suppurative otitis media at tertiary care hospital	Different complications can develop in spite of availability of higher antibiotics.	Parmar BD, Jha S, Sinha V, Chaudhury N, Dave G	10.18203/issn.2454-5929.ijohns20200146	2020
Study of bacterial pathogens causing chronic suppurative otitis media and the antibiotic susceptibility pattern of the isolates at a tertiary care center in Kochi	Chronic suppurative otitis media is a highly prevalent disease and commonly encountered in ENT OPD.	Geetha MN, Tanya TM, Bindhu V, Lancy J	10.18203/issn.2454-5929.ijohns20201288	2020
Detection of bio-film production among the most frequent bacterial isolates in cases of chronic suppurative otitis media: a cross-sectional study	The bacteria may gain entry to the middle ear through a chronic perforation.	Bhat P, Peer S, Yoganand R	10.20546/ijcmas.2020.906.167	2020
Bacteriological study of chronic suppurative otitis media	*Pseudomonas aeruginosa *are the most common isolated bacteria in chronic suppurative otitis media.	Hadi AA, Khammas AH, Alsaeed WM	10.26505/djm.19015680920	2020
Suppurative otitis media in Angola: clinical and demographic features	Patients with otorrhoa in Angola were characterized by demographic and clinical findings.	Filipe M, Karppinen M, Kuatoko P, Reimer Å, Riesbeck K, Pelkonen T	10.1111/tmi.13466	2020
Clinico-microbial profile of Otomycosis in discharging otitis media in tertiary care hospital	Acute suppurative otitis media is a major otolarynological problem in India commonly seen in children and adults.	Wagh K, Ghule S	10.18231/2394-5478.2018.0046	2020
Microbiology and antibiotic sensitivity of uncomplicated chronic suppurative otitis media at Dr. George Mukhari Academic Hospital, South Africa	The active form of chronic suppurative otitis media persists into adulthood in a very small percentage of the affected population.	Kotu M, Olwoch IP	10.18203/ISSN.2454-5929.IJOHNS20211177	2021
Unilateral chronic suppurative otitis media: effect of nasal septal deviation	Nasal septal deviation is commonly found in patients with unilateral chronic suppurative otitis media.	Ali AA, Majeed N	10.21474/IJAR01/12564	2021
Microorganisms and culture and sensitivity pattern in chronic suppurative otitis media in a tertiary care hospital	*Staphylococcus aureus* and *Pseudomonas aeruginosa *were the most common organisms responsible for chronic suppurative otitis media in our study.	Shrestha KS, Madhup SK, Shrestha BL, Pokharel M, Dhakal A	10.3126/jcmsn.v17i3.39719	2021
The study of bacteriological agents of chronic suppurative otitis media aerobic culture and antibiotic sensitivity testing pattern at a tertiary care hospital in and around Rajamundry: A cross-sectional study	Antimicrobials like imipenem, piperacillin, and quinolones are effective against most cases of chronic suppurative otitis media.	Shyamala R, Reddy PS	10.17511/jopm.2021.i04.09	2021
Role of 1.5% acetic acid irrigation and medical management in chronic persistent suppurative otitis media	Ear, nose, and throat infections are common clinical problems occurring in the general population.	Bhavya K, Sowmya L, Teja	10.18203/ISSN.2454-5929.IJOHNS20212124	2021
Bacterial agents causing chronic suppurative otitis media	*S. aureus *has been identified as the leading pathogen in chronic otitis media.	Jhajharia R	10.32553/ijmbs.v6i5.2539	2022
Case report: management of chronic supurative otitis media cholesteatoma type with recurrent brain abscess complication	Chronic suppurative otitis media with complications of cerebral abscess is treated with abscess drainage if the abscess size is >2 cm and followed by canal wall down mastoidectomy.	Ikhlas KA, Edward Y	10.19184/ams.v8i1.25338	2022
Complications of suppurative otitis media still occur in the 21st century	The most aggressive complication of suppurative otitis media is the malignant type.	Othman M, Hamza alshafae, Emaad alfasse	10.37375/sjms.v1i1.288	2022
Antimicrobial sensitivity profile of bacterial agents in chronic suppurative otitis media patients in Samawa city	The predominant aetiologies of otitis media were *Pseudomonas aeruginosa*, *Klebsiella pneumoniae*, *Proteus mirabilis*, and *Staphylococcus aureus*.	Habeeb AH	10.53730/ijhs.v6ns4.10448	2022
Methicillin-resistant *Staphylococcus aureus *(MRSA) infection in chronic suppurative otitis media.	The prevalence of MRSA otorrhoea in this study was relatively high.	Abubakar A, Isa A, Baba A, Yahaya M, Zailani A, Abdulmajid IY, Amina MA, Kodiya A	10.56167/jjms.2022.0301.05	2022
Evaluation of complications and management of chronic suppurative otitis media: a retrospective study	Patients should be given higher doses of intravenous antibiotics followed by mastoid surgery.	Mehwish H, Alina A, Arunandan K, Sheikh SA, Deepak R, Naveed A	10.53350/pjmhs22165461	2022
Case report: suppurative labyrinthitis induced by chronic suppurative otitis media	Suppurative labyrinthitis must not be overlooked and neglected.	Qianwen X, Yuzhong Z, Jingrong L, Jun Y, Qing Z	10.3389/fneur.2022.892045	2022
Clinical-morphological, intraoperative study of bone tissue in patients with chronic purulent otitis media	Bone tissue in patients with chronic suppurative otitis media is important for physicians, especially pediatricians, therapists.	Bashrullaevich MU, Abdullaevna KL	10.31435/rsglobal_ws/30062022/7823	2022

## References

[1] Teele DW, Klein JO, Rosner B (1989). Epidemiology of otitis media during the first seven years of life in children in greater Boston: a prospective, cohort study. J Infect Dis.

[2] Vergison A, Dagan R, Arguedas A (2010). Otitis media and its consequences: beyond the earache. Lancet Infect Dis.

[3] Fliss DM, Dagan R, Houri Z, Leiberman A (1990). Medical management of chronic suppurative otitis media without cholesteatoma in children. J Pediatr.

[4] Brook I (1995). Role of anaerobic bacteria in chronic otitis media and cholesteatoma. Int J Pediatr Otorhinolaryngol.

[5] Osma U, Cureoglu S, Hosoglu S (2000). The complications of chronic otitis media: report of 93 cases. J Laryngol Otol.

[6] Trimis G, Mostrou G, Lourida A, Prodromou F, Syriopoulou V, Theodoridou M (2003). Petrositis and cerebellar abscess complicating chronic otitis media. J Paediatr Child Health.

[7] Bluestone CD (2004). Studies in otitis media: Children's Hospital of Pittsburgh-University of Pittsburgh progress report-2004. Laryngoscope.

[8] Roland PS (2002). Chronic suppurative otitis media: a clinical overview. Ear Nose Throat J.

[9] Kenna MA, Rosane BA, Bluestone CD (1993). Medical management of chronic suppurative otitis media without cholesteatoma in children-update 1992. Am J Otol.

[10] Monasta L, Ronfani L, Marchetti F (2012). Burden of disease caused by otitis media: systematic review and global estimates. PLoS One.

[11] Arguedas A, Loaiza C, Herrera JF, Mohs E (1994). Antimicrobial therapy for children with chronic suppurative otitis media without cholesteatoma. Pediatr Infect Dis J.

[12] Morris PS (1998). Management of otitis media in a high risk population. Aust Fam Physician.

[13] Kenna MA, Bluestone CD, Reilly JS, Lusk RP (1986). Medical management of chronic suppurative otitis media without cholesteatoma in children. Laryngoscope.

[14] Vartiainen E (1992). Results of surgical treatment for chronic noncholesteatomatous otitis media in the pediatric population. Int J Pediatr Otorhinolaryngol.

[15] Bluestone CD (1998). Epidemiology and pathogenesis of chronic suppurative otitis media: implications for prevention and treatment. Int J Pediatr Otorhinolaryngol.

[16] Wintermeyer SM, Nahata MC (1994). Chronic suppurative otitis media. Ann Pharmacother.

[17] Kong K, Coates HL (2009). Natural history, definitions, risk factors and burden of otitis media. Med J Aust.

[18] van Hasselt P, van Kregten E (2002). Treatment of chronic suppurative otitis media with ofloxacin in hydroxypropyl methylcellulose ear drops: a clinical/bacteriological study in a rural area of Malawi. Int J Pediatr Otorhinolaryngol.

[19] Hurst DS, Denne CM (2020). The relation of allergy to eustachian tube dysfunction and the subsequent need for insertion of pressure equalization tubes. Ear Nose Throat J.

[20] (2023). WHO/CIBA Foundation Workshop: London UK, Impairment WHOP for the P of D and H, Foundation C: Prevention of hearing impairment from chronic otitis media : report of a WHO/CIBA Foundation Workshop, London, U.K., 19-21 November 1996. Foundation C: Prevention of hearing impairment from chronic otitis media : report of a WHO/CIBA Foundation Workshop, London, U.K., 19-21 November.

[21] Lewis K (2001). Riddle of biofilm resistance. Antimicrob Agents Chemother.

[22] Uddén F, Filipe M, Reimer Å (2018). Aerobic bacteria associated with chronic suppurative otitis media in Angola. Infect Dis Poverty.

[23] (2023). World Health Organization. Chronic suppurative otitis media: burden of illness and management options. https://apps.who.int/iris/handle/10665/42941.

[24] Stewart PS, Costerton JW (2001). Antibiotic resistance of bacteria in biofilms. Lancet.

[25] Schilder AG, Chonmaitree T, Cripps AW, Rosenfeld RM, Casselbrant ML, Haggard MP, Venekamp RP (2016). Otitis media. Nat Rev Dis Primers.

[26] Berry JA, Biedlingmaier JF, Whelan PJ (2000). In vitro resistance to bacterial biofilm formation on coated fluoroplastic tympanostomy tubes. Otolaryngol Head Neck Surg.

[27] Post JC, Stoodley P, Hall-Stoodley L, Ehrlich GD (2004). The role of biofilms in otolaryngologic infections. Curr Opin Otolaryngol Head Neck Surg.

[28] Verhoeff M, van der Veen EL, Rovers MM, Sanders EA, Schilder AG (2006). Chronic suppurative otitis media: a review. Int J Pediatr Otorhinolaryngol.

[29] Stenfors LE, Räisänen S (1991). Immunoglobulin-coated bacteria in effusions from secretory and chronic suppurative otitis media. Am J Otolaryngol.

[30] Rosenfeld RM, Kay D (2003). Natural history of untreated otitis media. Laryngoscope.

[31] Veenhoven R, Rijkers G, Schilder A, Adelmeijer J, Uiterwaal C, Kuis W, Sanders E (2004). Immunoglobulins in otitis-prone children. Pediatr Res.

[32] Gross S, Blaiss MS, Herrod HG (1992). Role of immunoglobulin subclasses and specific antibody determinations in the evaluation of recurrent infection in children. J Pediatr.

[33] Massa HM, Cripps AW, Lehmann D (2009). Otitis media: viruses, bacteria, biofilms and vaccines. Med J Aust.

[34] Berman S (1995). Otitis media in developing countries. Pediatrics.

